# Data on the involvement of endothelin-1 (ET-1) in the dysregulation of retinal veins

**DOI:** 10.1016/j.dib.2018.09.070

**Published:** 2018-09-29

**Authors:** Teruyo Kida, Josef Flammer, Hidehiro Oku, Katarzyna Konieczka, Seita Morishita, Taeko Horie, Tsunehiko Ikeda

**Affiliations:** a Department of Ophthalmology, Osaka Medical College, Takatsuki, Japan; b Department of Ophthalmology, University of Basel, Basel, Switzerland; c Department of Ophthalmology, Osaka Kaisei Hospital, Osaka, Japan

## Abstract

Retinal vein occlusion (RVO) is a common vascular disease of the retina; however, the pathogenesis of RVO is still unclear. Branch RVO (BRVO) commonly occurs at the arteriovenous crossing and it was formerly believed that the diseased artery mechanically compresses the vein. However, it has been reported that the retinal vein runs deep beneath the artery at the arteriovenous crossing in eyes with an arterial overcrossing, and the venous lumen often appears to be preserved, even at the arteriovenous crossing, as shown by optical coherence tomography. Paques et al. [1] found venous nicking without arteriovenous contact using adaptive optics imaging. Thus, we investigated the potential role of a dysregulation of the retinal vein.

While the pathogenesis of retinal vein occlusion (RVO) is still unclear, systemic hypertension and increased level of endothelin-1 (ET-1) are known risk factors (Flammer and Konieczka, 2015) [2]. We focused on the behavior of retinal veins in spontaneous hypertensive rats (SHR). Then, one of the retinal veins became exceptionally constricted and was nearly occluded (Fig. 1), and the chorioretinal blood flow significantly decreased in the retinas of SHRs following the intravenous injection of ET-1. In addition, immunoreactivity to ET-A receptor was higher in SHR retinas than in control (WKY; Wistar Kyoto rat) retinas (Fig. 2). The protein levels of ET-A receptor and HIF-1 were also significantly higher in SHR retinas than in WKY retinas (Fig. 3). We observed vasoactivity of retinal veins; a retinal venous constriction (Kida et al., 2018) [3]. This supports the hypothesis that ET-1 can constrict retinal veins, thus increasing retinal venous pressure, and that ET-1 may even contribute to the pathogenesis of RVO.

**Specifications table**

**Table:** 

Subject area	*Pathophysiology*
More specific subject area	*Molecular biology, pathophysiology*
Type of data	*Text file, figure*
How data was acquired	*Microscope, IHC, western blot analysis, laser speckle flowgraphy (LSFG) (LSFG-Micro, Softcare, Fukuoka, Japan)*
Data format	*Raw, analyzed*
Experimental factors	*IHC stained retinal tissue, IHC stained flat mount retina, Chorioretinal blood flow, protein levels of ET-A and ET-B receptors and HIF-1 in retinal tissues*
Experimental features	*IHC images, Observed retinal veins and measured ocular blood flow, western blot analysis*
Data source location	*Ophthalmology, Osaka Medical College, Takatsuki, Japan*
Data accessibility	*The data is with this article* and Ref. [3].

**Value of the data**

• One of the retinal veins became exceptionally constricted and was nearly occluded, and the chorioretinal blood flow significantly decreased in the retinas of SHRs following the intravenous injection of ET-1.
• The chorioretinal blood flow decreased after the intravenous injection of ET-1 in all WKYs and SHRs; however, there was a statistically significant difference in the decreases in blood flow from the baseline between WKYs (−7.3 ± 3.0%, mean ± S.D.) and SHRs (−17.3 ± 8.3%) (Student׳s *t*-test; *P* < 0.05).
• The immunoreactivity to ET-A receptor was significant in retinal vessels of the flat mount retina. In addition, it was significantly higher in SHR retinas than in WKY retinas.
• The protein levels of ET-A receptor and HIF-1 were also significantly higher in SHR retinas than in WKY retinas.
• The increase in plasma ET-1 was significantly greater in SHRs (1.15 ± 0.21 pg/mL, mean ± S.D.) than in WKYs (0.57 ± 0.12 pg/mL) (Student׳s *t*-test; *P* < 0.05; *n* = 5 each).

## 1 Data

**Fig. 1:**
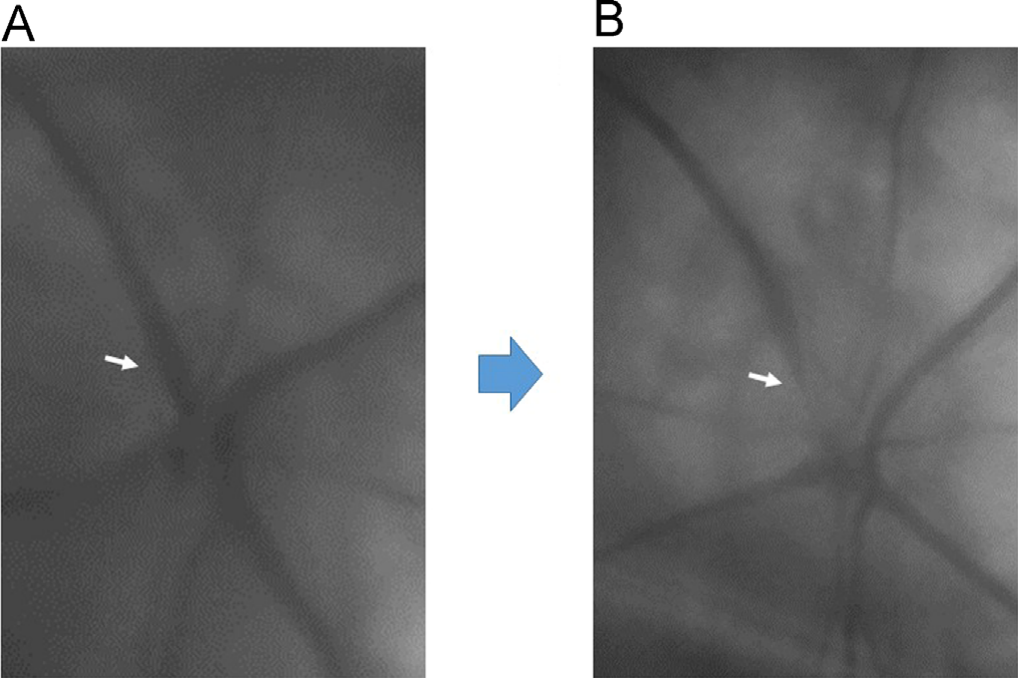
Fundus photos of optic nerve head (A and B) before and after the intravenous injection of ET-1 in SHRs. One of the retinal veins became exceptionally constricted and nearly occluded (arrow, B).

**Fig. 2:**
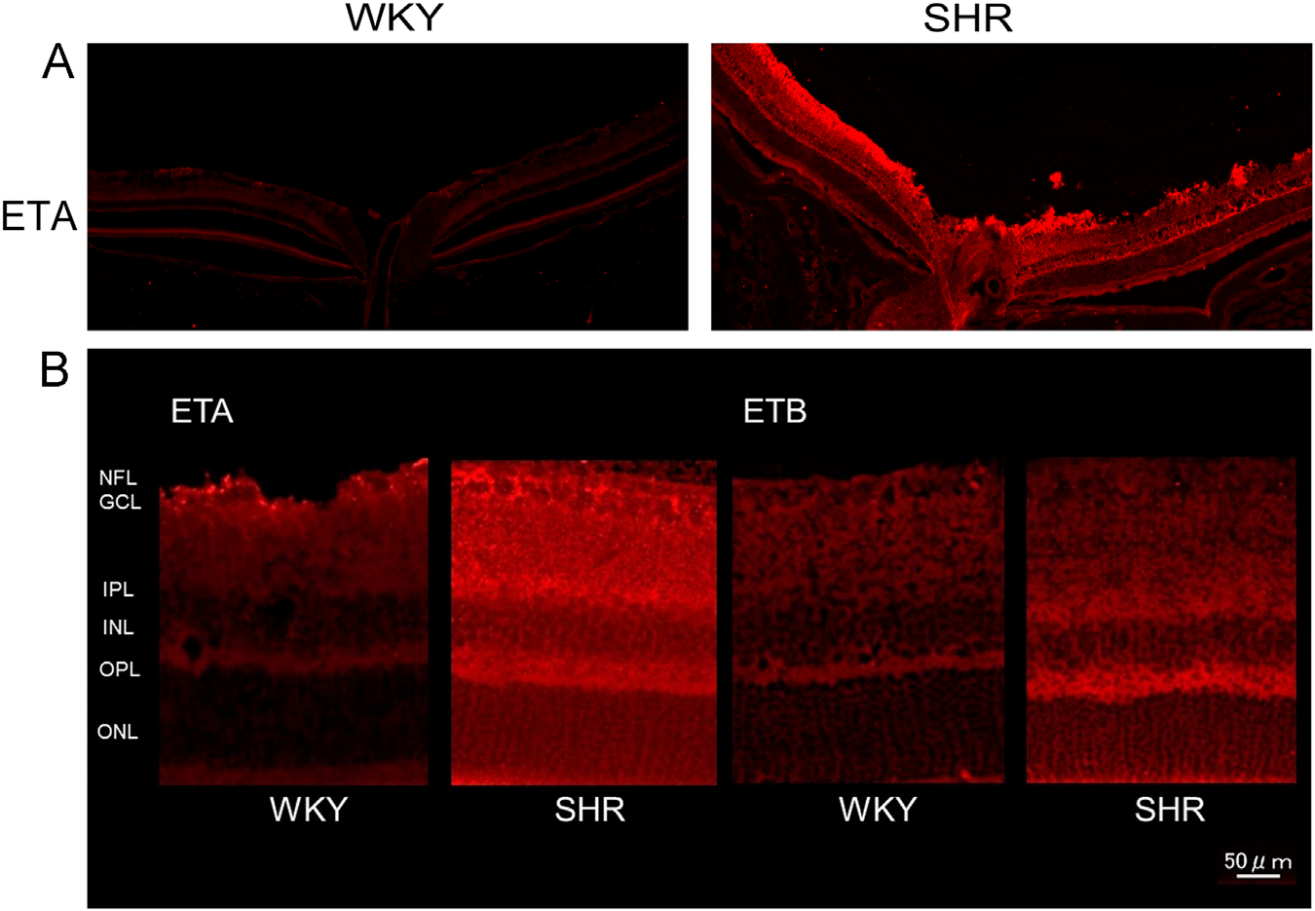
(A) Immnohistochemical-stained retina tissue sections at the level of the optic nerve head. The immunoreactivity to ET-A receptors were significantly higher in SHR retinas than in WKY retinas. (B) Immunohistochemical-stained retina sections of ET-A and ET-B receptors in WKY and SHR.

**Fig. 3:**
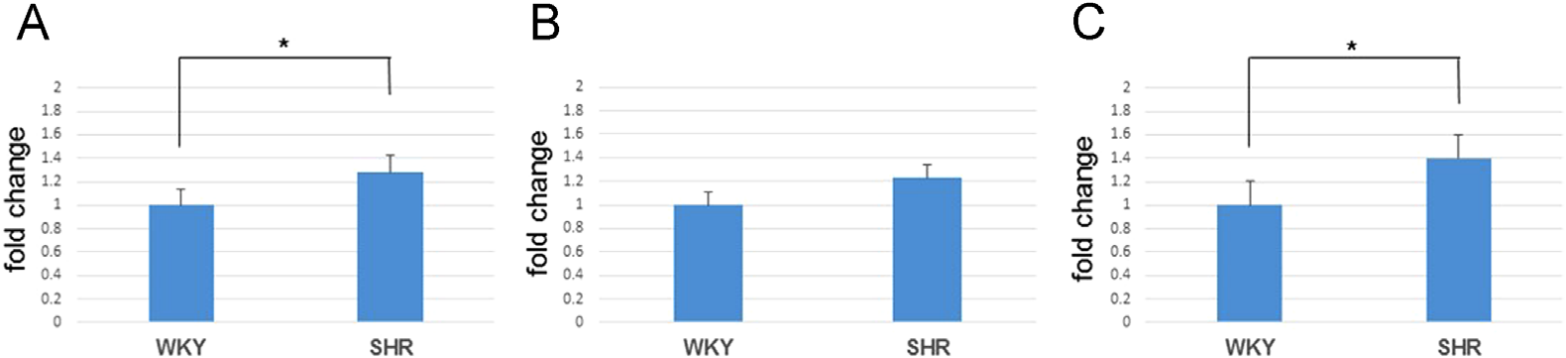
Protein levels of both types of ET receptors and HIF-1 by western blot. The protein levels of ET-A receptor (A) and HIF-1 (C) were significantly higher in SHR retinas than in WKY retinas (Student׳s *t*-test; *P* < 0.05; *n* = 5 each).

See Fig. 1, Fig. 2, Fig. 3.

## 2 Experimental design, materials and methods

To examine whether ET-1 was associated with the blood flow in the eyes of SHRs (9–11 weeks of age, *n* = 5), the chorioretinal blood flow in the rats was assessed using laser speckle flowgraphy (LSFG) (LSFG-Micro, Softcare, Fukuoka, Japan) before and after an intravenous injection of ET-1 (2 nmol/kg) under general anesthesia. The methods and principles of LSFG have been described in a previous study. LSFG imaging provides a relative index of blood velocity represented as MBR (mean blur rate), which is determined by analyzing the blurring of the speckle pattern formed through the interference of a laser that is scattered by the movement of blood cells. In addition, retinas from SHRs and age-matched normotensive Wistar-Kyoto rats (WKYs) (*n* = 5 each) were fixed by perfusion under deep anesthesia with a mixture of medetomidine, midazolam hydrochloride, and butorphanol tartrate. The retinal tissues were removed, and retinal sections were immunostained for the ET-A (1:500, Abcam plc, Cambridge, UK) and ET-B (1:500, Sigma-Aldrich, St. Louis, MO, USA) receptors. In addition, we performed immunohistochemical staining of flat mount retina. The retinal flat mounts were immunostained for ET-A, ET-B, and α-smooth muscle actin (SMA) (1:500, Sigma-Aldrich, St. Louis, MO, USA). The processed sections were photographed with a fluorescent microscope (BZ-X700, Keyence, Osaka, Japan). The protein levels of both ET-1 receptors (1:1000) and hypoxia-inducible factor 1 (HIF-1) (1:1000, Santa Cruz, Dallas, TX, USA) in the retinal tissues were also determined by western blot analysis. Samples containing 20 μg of protein were run on 7.5% to 10% SDS-PAGE gels and electroblotted onto polyvinylidene difluoride membranes. In addition, the plasma ET-1 concentrations in WKYs and SHRs were measured by ELISA (Quantikine^®^ELISA Endothelin-1 Immunoassay, R&D Systems, Inc., Minneapolis, MN, U.S.A.). Our experimental protocols conformed to guidelines in Animal Research: Reporting in vivo Experiments (ARRIVE) and were approved by the Osaka Medical College Committee on the Use and Care of Animals (approval number: 29097).

## References

[1] Paques M., Brolly A., Benesty J. (2015). Venous nicking without arteriovenous contact: the role of the arteriolar microenvironment in arteriovenous nickings. JAMA Ophthalmol..

[2] Flammer J., Konieczka K. (2015). Retinal venous pressure: the role of endothelin. EPMA J..

[3] Kida T., Flammer J., Oku H. (2018). Vasoactivity of retinal veins: a potential involvement of endothelin-1 (ET-1) in the pathogenesis of retinal vein occlusion (RVO). Exp. Eye Res..

